# Adaptive response of resistant cancer cells to chemotherapy

**DOI:** 10.20892/j.issn.2095-3941.2020.0005

**Published:** 2020-12-15

**Authors:** Yi-Jye Chern, Isabella T. Tai

**Affiliations:** 1 Division of Gastroenterology, Department of Medicine, University of British Columbia, Vancouver, British Columbia V5Z1L3, Canada; 2 Michael Smith Genome Sciences Center, British Columbia Cancer Agency, Vancouver, British Columbia V5Z1L3, Canada

**Keywords:** Cancer, adaptive response, chemoresistance, chemotherapy, autophagy, ER stress signaling, senescence

## Abstract

Despite advances in cancer therapeutics and the integration of personalized medicine, the development of chemoresistance in many patients remains a significant contributing factor to cancer mortality. Upon treatment with chemotherapeutics, the disruption of homeostasis in cancer cells triggers the adaptive response which has emerged as a key resistance mechanism. In this review, we summarize the mechanistic studies investigating the three major components of the adaptive response, autophagy, endoplasmic reticulum (ER) stress signaling, and senescence, in response to cancer chemotherapy. We will discuss the development of potential cancer therapeutic strategies in the context of these adaptive resistance mechanisms, with the goal of stimulating research that may facilitate the development of effective cancer therapy.

## Introduction

The high prevalence and mortality rate of cancer is a major burden to human health worldwide^1^. Unfortunately, despite extensive efforts and advances in cancer research, only a slight decrease in the cancer death rate has been observed^2^. The limited efficacy of chemotherapy, which is one of the principal modes of cancer treatment, is considered to be a major hindrance to our ability to effectively treat and manage the disease. In order to improve current cancer therapies and to develop novel treatment strategies, a better understanding of the mechanisms underlying the limitations of chemotherapy is urgently needed.

The factors contributing to the limited success of chemotherapy are complicated and multifactorial, and our inability to accurately predict how cancer patients will respond to drug treatment is significant. Recent technological advances have facilitated the molecular understanding of cancers and the identification of targets for therapeutic interventions^3^ *via* computational analysis. However, these methods are limited by intra-tumor heterogeneity, as characteristics of the major tumor cell type may not necessarily predict the features of mixed populations^4^. Furthermore, rare mutations in tumors are often undetected due to the limitations of sequencing technology. For example, sequencing at the initiation of treatment may fail to detect cancer cells harboring mutations that confer resistance to chemotherapeutics, such as mutations in *KRAS*. Over the course of treatment, selective pressure results in the expansion and proliferation of drug-resistant cells^5^. In addition, therapeutics may have differing levels of efficacy and toxicity in individuals with varied genetic backgrounds. For instance, mutations in *TP53* have been shown to contribute to the risk of treatment failure in patients with relapsed childhood acute lymphoblastic leukemia^6^. These complications exemplify the need for individualized and tailored cancer treatment in order to maximize efficacy and minimize unwanted side effects. However, the field of personalized medicine is still under development, and a myriad of obstacles must be overcome before it can be applied in clinics^7^.

The development of drug resistance in cancer cells is arguably one of the most challenging factors limiting the success of chemotherapy. Chemoresistance can be broadly categorized into two types: (1) intrinsic resistance and (2) acquired resistance. The two groups differ in the origin of resistance: intrinsic resistance pre-exists within the cancer (cancer cells are resistant to initial treatments by chemotherapeutic agents) while acquired resistance emerges in response to treatment (resistance develops in cancer cells after initiation of treatment with chemotherapeutic agents). However, they share common mechanisms of resistance including reduced drug transport, altered drug targets, metabolic adaptations, dysregulation of DNA damage repair pathways, defective apoptotic signaling, activation of pro-survival signaling, pro-tumorigenic microenvironments, and cellular adaptive responses^8^.

**Figure 1:**
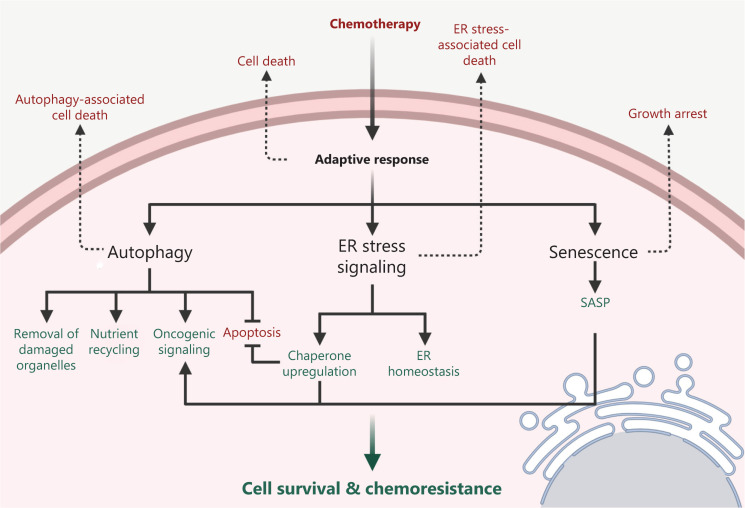
Mechanisms of adaptive response induced by chemotherapy in the cancer cells. The administration of chemotherapy may lead to the disruption of cellular homeostasis followed by the activation of multiple signaling pathways including autophagy, endoplasmic reticulum (ER) stress signaling, and senescence. Cellular homeostasis may be restored by autophagy through the removal of damaged organelles and the recycling of nutrients, or by the induction of ER stress signaling through the recovery of ER homeostasis and the upregulation of chaperones. Some oncogenic signaling pathways can also be activated through the autophagy, ER stress signaling, or in the senescent cells with the senescence-associated secretory phenotype (SASP), thereby promoting cell survival and chemoresistance. On the contrary, depending on the type, intensity, and duration of the therapy-associated stress, adaptive response signaling may fail to be activated and eventually lead to cell death or growth arrest.

The cellular response to stress can lead to either the activation of cell death pathways or the adaptive response that maintains the survival of the cells. The adaptive response is the ability of a cell, tissue, or organism to better resist stress damage by prior exposure to a sublethal stress, including changes in temperature, oxygen tension, redox potential, extracellular signals, and chemical insults such as chemotherapeutic drugs^9,10^. During the adaptive process, cells undergo dramatic metabolic and physiological adaptations to prevent cellular damage and to maintain homeostasis. This is accomplished through the concerted action of diverse molecular signaling including autophagy, ER stress signaling, and senescence^10^. Accumulating evidence has revealed that these adaptive responses are crucial for tumorigenesis, tumor survival, and tumor progression^11–13^. This review will focus on the mechanisms by which autophagy, ER stress signaling, and senescence promote cell survival and contribute to the resistance in cancer cells exposed to targeted therapies (**Table 1**) and chemotherapeutic drugs (**Figure 1**).

**Table 1:** List of clinical targeted therapeutic agents inducing an adaptive response in cancer cells

Adaptive response	Drugs (generic name)	Trade name	Drug type	Target protein	Cancer	Reference
**Autophagy**	Afatinib	Gilotrif	Tyrosine kinase inhibitor	EGFR	NSCLC	^204^
	Bevacizumab	Avastin	Monoclonal antibody	VEGF-A	CRC	^30^
					GBM	^205^
	Bortezomib	Velcade	Proteasome inhibitor	26S proteasome	Breast cancer	^97,206^
	Cetuximab	Erbitux	Fv (variable, antigen-binding) regions of monoclonal antibody	EGFR	Lung cancer, CRC	^59^
	Dasatinib	Sprycel	Tyrosine kinase inhibitor	BCR/ABL, Src family	NSCLC	^207^
	Gefitinib	Iressa	Tyrosine kinase inhibitor	EGFR	Breast cancer	^65^
	Idelalisib	Zydelig	PI3K inhibitor	P110 delta	CML	^98^
	Lapatinib	Tykerb	Tyrosine kinase inhibitor	EGFR, HER2	Breast cancer	^208^
					HCC	^209^
	Osimertinib	Tagrisso	Tyrosine kinase inhibitor	EGFR	NSCLC	^210^
	Sorafenib	Nexavar	Tyrosine kinase inhibitor	Raf, PDGF, VEGFR2/3, Kit	RCC	^211^
	Sunitinib	Sutent	Tyrosine kinase inhibitor	PDGFR, VEGFR, KIT	HCC	^212^
					mRCC	^213^
					PanNET	^214^
	Trametinib	Mekinist	MEK kinase inhibitor	MEK1/2	Melanoma	^215^
					Leukemia	^216^
	Trastuzumab	Herceptin	Monoclonal antibody	HER2	Breast cancer	^217–219^
	Vemurafenib	Zelboraf	Competitive kinase inhibitor	BRAF (V600E)	Melanoma	^220^
	Vismodegib	Erivedge	Cyclopamine-competitive antagonist	SMO	CML	^221^
**ER stress**	Bortezomib	Velcade	Proteasome inhibitor	26S proteasome	Breast cancer	^97^
	Trastuzumab	Herceptin	Monoclonal antibody	HER2	Breast cancer	^222^
**SASP**	Sunitinib	Sutent	Tyrosine kinase inhibitor	PDGFR, VEGFR, KIT	Breast cancer	^193^

EGFR, epidermal growth factor receptor; VEGF-A, vascular endothelial growth actor A; HER2, human epidermal growth factor receptor 2; MEK1/2, mitogen-activated protein kinase kinase 1/2; VEGFR, vascular endothelial growth factor receptor; SMO, smoothened; NSCLC, non-small cell lung cancer; CRC, colorectal cancer; GBM, glioblastoma; CML, chronic myeloid leukemia; HCC, hepatocellular carcinoma; mRCC, metastatic renal cell carcinoma; PanNET, pancreatic neuroendocrine tumors.

## Autophagy

Macroautophagy (hereafter denoted as autophagy) is a dynamic process in which double-membrane vesicles, or autophagosomes, are formed to sequester cytoplasm or organelles. Autophagosomes are then targeted to lysosomes where the autophagosomal cargo is degraded and recycled for the needs of the cell. Autophagy is an important mechanism for maintaining intracellular homeostasis. Unfolded proteins or dysfunctional mitochondria can be eliminated through selective autophagy, thereby preventing the excessive production of reactive oxygen species (ROS), which cause genome instability and elicit tumorigenesis. In addition, autophagy is considered a distinct type of programmed cell death (type II programmed cell death).

Due to its involvement in cell death, autophagy may be a tumor-suppressive mechanism. Indeed, the monoallelic deletion of *BECN1* (encoding Beclin1), a gene essential for autophagy, is observed in 40%–75% of human ovarian, breast, and prostate cancer tissues^14^. The heterozygous disruption of *BECN1* has also been shown to promote tumorigenesis in a mouse model^14^. However, there is evidence demonstrating that autophagy actually supports tumorigenesis in some settings and may promote tumor growth and cancer cell survival in established tumors^15,16^. The tumor-promoting activity of autophagy may partly come from its ability to restore nutritional and oxidative homeostasis under stress conditions including hypoxia, tumor acidosis, extracellular matrix detachment, and oncogene-induced transformation. Importantly, several studies have demonstrated that inhibition of autophagy may be a therapeutic strategy for cancer patients of certain stages^16,17^.

The paradoxical involvement of autophagy in both tumor suppression and progression is also in line with its complex role in the cellular response to chemotherapy. Upregulation of autophagy has been found in drug-resistant cells and has been shown to be a protective mechanism against therapeutic stress^18^ (**Table 2**). Alternatively, enhancing autophagy could potentially lead to autophagy-associated cell death, synergizing with chemotherapy to suppress tumor growth. As a context-dependent mediator of chemotherapeutic responses, the role of autophagy is influenced by different factors such as the tumor stroma and oncogenic signaling in cancer cells, as detailed below.

**Table 2:** List of clinical chemotherapeutic drugs inducing autophagy in cancer cells

Drug (generic name)	Trade name	Drug type	Cancer	Reference
5-Fluorouracil	Adrucil	Antimetabolite	CRC	^83,223–225^
			HCC	^82^
Asparaginase	Elspar/Kidrolase	Enzyme	ALL	^226^
Cisplatin	Platinol	Alkylating agent	Ovarian cancer	^227^
			Esophageal cancer	^228^
			Desmoid tumors	^229^
			NSCLC	^230^
			HGSOC	^231^
Dexamethasone	Decadron	Glucocorticosteroid	Lymphoid malignancy	^232^
Docetaxel	Taxotere	Plant alkaloid/taxane/antimicrotubule agent	Prostate cancer	^233^
Doxorubicin	Adriamycin/Rubex	Anthracycline antibiotic	Leukemia	^234^
			Multiple myeloma	^235^
			Melanoma	^236^
			CRPC	^237^
Enzalutamide	Xtandi	Anti-androgen	Prostate cancer	^238^
Epirubicin	Ellence	Anthracycline antibiotic	TNBC	^239^
			Breast cancer	^240^
			HCC	^241^
Etoposide	Etopophos	Anthracycline antibiotic	SCLC	^242^
Gemcitabine	Gemzar	Antimetabolite	Pancreatic cancer	^243^
Irinotecan (CPT-11)	Camptosar	Plant alkaloid/topoisomerase I inhibitor	CRC	^244^
Oxaliplatin	Eloxatin	Alkylating agent	CRC	^30,245^
			HCC	^82^
Paclitaxel	Taxol/Onxal	Plant alkaloid/taxane/antimicrotubule agent	Cervical cancer	^246^
			Ovarian cancer	^247^
			Breast cancer	^248^
Pemetrexed	Alimta	Antimetabolite	HCC	^249^
Vincristine	Oncovin	Plant alkaloid	Leukemia	^234^

CRC, colorectal cancer; HCC, hepatocellular carcinoma; ALL, acute lymphocytic leukemia; NSCLC, non-small cell lung cancer; CRPC, castration-resistant prostate cancer; TNBC, triple negative breast cancer; SCLC, small cell lung cancer.

## Autophagy under the influence of the tumor stroma

### Endothelial cells

The tumor stroma includes (i) the extracellular matrix, (ii) mesenchymal cells such as fibroblast and myofibroblast/cancer-associated fibroblasts (CAFs), (iii) blood and lymphoid vessels, and (iv) nerve and inflammatory cells. It is a complex, three-dimensional compartment that surrounds the parenchyma and influences tumor growth, metastasis, and therapeutic responses^19,20^. Anti-angiogenic cancer therapies directly target the tumor stroma by destroying the tumor vasculature, thereby depriving the tumor of oxygen and nutrients^21^. Although preclinical and clinical trials afford demonstrable efficacy of anti-angiogenic therapy, the benefits are at best transitory and are followed by a restoration of tumor growth and progression^22^. Multiple resistance mechanisms to anti-angiogenic therapy have been proposed and recent studies have indicated that the activation of autophagy is one of them^23,24^. Hypoxia, resulting from the anti-angiogenic therapy-mediated devascularization of tumors, was shown to activate autophagy *via* pathways controlled by hypoxia-inducible factor 1α (HIF-1α) and adenosine monophosphate (AMP)-activated protein kinase (AMPK)^24^. HIF-1α activation led to the expression of genes encoding the Bcl-2 homology 3 (BH3)-only proteins BCL2/adenovirus E1B 19-kDa-interacting protein 3 (*BNIP3*) and BNIP3-like (BNIP3L). The atypical BH3 domains of these proteins can induce autophagy through disruption of the Bcl-2–Beclin1 complex, thereby releasing Beclin1, a major autophagy activator^25,26^. AMPK has been shown to activate Unc-51-like kinase 1 (ULK1), a key initiator of autophagy, through phosphorylation and inhibition of mammalian target of rapamycin complex 1 (mTORC1) activity, leading to the induction of autophagy in cancer cells^27^.

Autophagy promotes tumor cell survival under anti-angiogenic treatment by clearing damaged organelles, reducing oxidative metabolism, and providing nutrients when blood perfusion is limited^25,26^. Preclinical studies have shown that autophagy-related genes were upregulated upon treatment with the vascular endothelial growth factor (VEGF)-neutralizing antibody bevacizumab in glioblastoma, hepatocarcinoma, and colon cancer^28–30^. In addition, in the glioblastoma xenograft model, *in vivo* targeting of the essential autophagy gene *ATG7* resulted in tumor suppression when combined with bevacizumab^28^. Importantly, tumors from glioblastoma patients resistant to bevacizumab were shown to have increased regions of hypoxia and elevated levels of *BNIP3* expression compared with the pretreatment specimens from the same patients^28^. Furthermore, the combination of the autophagy inhibitor chloroquine with bevacizumab significantly increased apoptosis of cancer cells under hypoxia, suggesting that the combinatorial treatment may be effective in curbing resistance to anti-angiogenic therapy^29,30^.

Interestingly, recent studies have reported that the induction of autophagy in both tumor and endothelial cells may negatively regulate angiogenesis^31–34^. While the exact mechanisms remain to be elucidated, some studies indicate that it may be in part due to the degradation of angiogenic factors through autophagy. For example, gastrin-releasing peptide (GRP) is a gut neuropeptide that promotes endothelial cell proliferation and stimulates angiogenesis in various cancers^35^. Following the induction of autophagy, enhanced degradation of GRP and subsequent inhibition of endothelial cell proliferation and tubule formation were observed^32^. However, it remains to be seen whether the inhibition of angiogenesis by treatment-induced autophagy will exacerbate hypoxic conditions of the tumor microenvironment, thereby establishing a vicious cycle that further increases chemoresistance.

### Fibroblast

Fibroblasts that acquire activated phenotypes in response to the pro-fibrotic factors secreted by cancer cells, such as transforming growth factor-β (TGF-β), platelet-derived growth factor (PDGF), and fibroblast growth factor 2 (FGF2), are termed myoblasts or CAFs^19^. CAFs have been implicated in tumorigenesis, tumor progression, and chemoresistance in several cancers^36,37^. Their role and mechanisms in cancers have been recently reviewed^38,39^. The loss of caveolin-1 (Cav-1) in CAFs is associated with poor prognosis and tamoxifen resistance in human breast cancer patients^40,41^. Martinez-Outschoorn et al.^42,43^ demonstrated that breast cancer cells induced high oxidative stress and activated autophagy in CAFs, leading to their autophagic degradation of Cav-1 *via* HIF-1α and nuclear factor kappa B (NFκB) activation. Proteomic analysis revealed that the loss of Cav-1 in the stroma further elevated oxidative stress in CAFs and promoted their transformation^44^. CAFs have also recently been shown to increase the cell viability of tongue squamous cell carcinoma treated with cisplatin^45^. In addition, autophagic CAFs have been shown to contribute to chemoresistance by upregulating the expression of TP53-induced glycolysis regulatory phosphatase (TIGAR) in adjacent cancer cells^46^. TIGAR is a novel p53-inducible protein that has been shown to decrease intracellular ROS levels and reduce the sensitivity of the cell to p53 and other ROS-associated apoptotic signals induced by drugs^43,46^.

## Autophagy under the influence of oncogenic signaling

Aberrant activation of signaling pathways contributes to tumorigenesis and tumor progression and mediates the cellular response to chemotherapy in many human malignancies. Several studies have demonstrated that cancer cells utilize autophagy to cope with oncogenic stress^47^. The links between autophagy and oncogenic signaling pathways, and how this interplay regulates chemoresistance in cancer cells, are gradually emerging. Epidermal growth factor receptor (EGFR) is a receptor tyrosine kinase that is over-activated in most epithelial cancers due to *EGFR* mutations and/or overexpression^48^. Small-molecule receptor tyrosine kinase inhibitors (TKI), such as erlotinib and gefitinib, have been shown to be effective in blocking EGFR activity, especially in non-small cell lung cancers (NSCLCs) with an in-frame deletion in exon 19 or the single base substitution resulting in an L858R mutation^49,50^. However, approximately 30% of NSCLC patients demonstrated intrinsic resistance to TKI therapy, partly explained by the clonal MET amplification^51,52^. Those responding to therapy inevitably developed drug resistance *via* various mechanisms such as the T790M secondary mutation^50^. Recently, the non-genetic resistance through the activation of Aurora A and Aurora B has been reported to be associated with acquired resistance to EGFR TKIs^53,54^, and the treatment with Aurora kinase inhibitors could enhance and prolong the EGFR inhibitor response in preclinical models^53,54^. Studies have also indicated that autophagy is implicated in TKI resistance. In lung cancer cell lines with either wild-type or mutant *EGFR*, treatment with erlotinib and gefitinib induced autophagy, and the degree of induction was greater in resistant cells, suggesting that autophagy is involved in both innate and acquired resistance to EGFR-target therapy^55–58^. In addition, higher basal autophagy levels have been demonstrated in geftinib- resistant cell lines compared to parental cells^57^. Similarly, the EGFR-blocking antibody cetuximab has been shown to induce autophagy in lung cancer cell lines, promoting cell survival^59,60^. Treatment with TKIs and cetuximab was shown to induce autophagy through the inhibition of the class I phosphatidylinositol 3-kinase (PtdIns3K)/protein kinase B (Akt)/mTOR pathway as well as activating the class III PtdIns3K (hVps34)/Beclin1 autophagic pathway^55,60^. Of note, glioblastoma patients expressing *EGFRvIII*, an *EGFR* mutation variant associated with therapy resistance, were recently found to be particularly responsive to treatment with the autophagy inhibitor chloroquine^61^, suggesting the involvement of autophagy in the *EGFRvIII^+^* tumors.

The mechanisms by which TKI inhibitors induce resistance by regulating autophagy are emerging. As mentioned already, TKI treatment can induce autophagy activation through its inhibition of the phosphatidylinositol-3-kinase (PI3K)/Akt/mTOR pathway. Some studies have also demonstrated that EGFR itself directly interacts with the core autophagy machinery, and the treatment of TKI may disrupt this interaction, leading to the activation of autophagy and cell survival. For example, Wei et al.^62^ demonstrated that active EGFR mediates Beclin1 phosphorylation, leading to its inhibition and decreased autophagy. TKI therapy, however, disrupts Beclin1 phosphorylation, restoring autophagy. Furthermore, K721A kinase-dead (KD) EGFR, which mimics the blocking of EGFR by TKIs, was able to cooperate with lysosomal-associated transmembrane protein 4B (LAPTM4B) and Sec5 at endosomes to cause the disassociation of the autophagic inhibitor Rubicon from Beclin1, thereby initiating autophagy^63^. These data suggest that EGFR inhibition may directly promote autophagy, and that co-targeting EGFR and autophagy may be a promising approach for overcoming chemoresistance under TKI treatment. Indeed, some preclinical studies have reported synergism between the autophagy inhibitors chloroquine or hydroxychloroquine and EGFR TKIs in NSCLC, breast cancer, and glioblastoma^55,56,64,65^.

Another common mutation that can be targeted therapeutically is the V600E mutation in the v-RAF murine sarcoma viral oncogene homolog B1 (BRAF), which constitutively activates the BRAF kinase, resulting in sustained extracellular signal-related kinase (ERK) signaling and increased tumor growth^66^. In melanoma cells with *BRAF*^V600E^, hyperactivation of ERK induced higher levels of autophagy^67^. In BRAF^V600E^-driven lung tumors in mice, autophagy was essential for mitochondria metabolism and tumor growth, and deletion of an essential autophagy gene prolonged survival compared to the wild-type control mice^68^. The defect in mitochondrial respiration in autophagy-deficient *BRAF^V600E^* cancer cells was shown to be rescued by the addition of exogenous glutamine, which suggests that autophagy promotes cancer survival in part through sustaining mitochondrial metabolism and by providing essential amino acids. This phenomenon is termed “autophagy addiction”^69^. Furthermore, the activation of autophagy may also promote drug resistance in cancer cells treated with the BRAF inhibitor (BRAFi) vemurafenib, as the biopsies from BRAFi-resistant melanoma patients exhibited increased levels of autophagy compared with baseline^70^. Importantly, both melanoma and brain tumor cells with *BRAF*^V600E^ (but not wild type) displayed synergy when chloroquine was combined with vemurafenib, and tumor regression was observed in BRAFi-resistant xenografts and cancer patients, suggesting that combination therapies may delay the acquisition of resistance and improve patient outcomes^70,71^. Li et al.^72^ demonstrated that treatment with BRAFi induced autophagy *via* a transcriptional program coordinating lysosomal biogenesis and function mediated by transcription factor EB (TFEB), providing a clue to the mechanism of BRAFi-induced autophagy.

Kirsten rat sarcoma viral oncogene homolog (K-Ras) is an upstream regulator of BRAF and the mutant *KRAS* is associated with aggressive cancer phenotypes and poor patient prognosis^73^. The expression of *KRAS*^V12^ has been shown to upregulate basal autophagy, which was required to maintain the pool of functional mitochondria needed to deal with the high levels of oxidative phosphorylation and ROS in K-Ras-driven tumors^15,74^. Ablation of autophagy was also shown to decrease K-Ras-mediated adhesion-independent transformation, proliferation, and cell survival in several *KRAS*^G12D^-driven cancers, such as pancreatic, breast, and lung cancer cells^15,74–77^.

## Autophagy and non-coding RNA

The advancements in genomic technology have altered our perception of non-coding RNAs (ncRNAs) from non-functional (sometimes referred to as junk) to regulatory molecules that modulate cellular processes and play important roles in diseases like cancer^78,79^. ncRNA species include microRNAs (miRNAs), circular RNAs (circRNAs), and long ncRNAs (lncRNAs)^79^. Recent studies have demonstrated that lncRNAs may influence the sensitivity of cancer cells to chemotherapy *via* autophagy^80,81^. For example, both lncRNAs HULC (highly upregulated in liver cancer) and H19 were found to be associated with 5-fluorouracil resistance *via* their modulation of autophagy regulator sirtuin 1 (SIRT1)^82,83^. Their expression levels were found to increase in liver and colorectal cancers, respectively, and were found to be associated with cancer recurrence^83^. Similarly, gallbladder cancer drug resistance-associated lncRNA1 (GBCDRlnc1) was found to be upregulated in both gallbladder cancer tissues and doxorubicin-resistant gallbladder cancer cells. Knockdown of GBCDRlnc1 may increase the sensitivity of Dox-resistant cancer cells *via* inhibiting autophagy^84^. Importantly, lncRNAs have also been found to increase chemoresistance by modulating the expression of miRNAs and thus the downstream autophagic signaling. For instance, metastasis-associated lung adenocarcinoma transcript 1 (MALAT1), a ncRNA known to be a regulator of metastasis in NSCLC^85^, has recently been shown to promote autophagy and increase chemoresistance by miR-124 downregulation and miR-23b-3p sequestration^86,87^. On the other hand, lncRNAs have also been observed to reduce chemoresistance in cancer cells by suppressing autophagy. For instance, lncRNA growth arrest-specific 5 (GAS5) was found to increase the sensitivity to cisplatin in glioma cells by activating mTOR signaling, thereby inhibiting autophagy^88^.

Taken together, these studies suggest an emerging role of lncRNA in autophagy regulation and chemoresistance in cancer cells. The lncRNA profiles of cancer patients should be taken into consideration when designing therapeutic strategies, especially *via* autophagy inhibition.

## ER stress signaling

The ER is a perinuclear, cytosolic organelle which is required for cell survival and normal cellular function. The functions of ER include intracellular calcium homeostasis, lipid biosynthesis and protein folding, modification, and secretion. Disturbances, such as nutrient deprivation, hypoxia, ER Ca^2+^ depletion, and oxidative stress can impair glycosylation or protein disulfide bond formation, leading to the accumulation of unfolded proteins in the ER, triggering an evolutionarily conserved protein quality control mechanism termed unfolded protein response (UPR) or ER stress signaling pathways^89^. Canonical UPR signaling includes three pathways, each mediated by a different ER stress sensor: protein kinase RNA-like ER kinase (PERK), inositol-requiring kinase 1α (IRE1α), and the activating transcription factor 6 (ATF6). Glucose-regulated protein, 78 kDa (GRP78), a major ER chaperone, acts as a master regulator of the UPR through direct binding to all three sensors, keeping them in an inactive form. Upon ER stress, GRP78 preferentially binds the accumulating misfolded proteins, leading to the activation of the three sensors and the transduction of UPR signals across the ER membrane to the cytosol and ultimately the nucleus^90^. Triggering UPR can lead to cell survival or cell death, depending on the cellular context, the intensity of stress, and the length of exposure. The function of UPR is to restore ER homeostasis, mainly by suppressing global mRNA translation *via* PERK-mediated phosphorylation of eukaryotic translation initiation factor 2α (eIF2α), leading to reduced influx of new proteins into the ER. As the stress continues, adaptive mechanisms become activated. The adaptive response primarily involves the activation of autophagy and transcriptional programs that induce expression of genes for enhancing ER protein folding and ER-assisted degradation (ERAD). ERAD facilitates removal of the unfolded proteins in the ER and export to the cytosol for degradation^91^. However, when adaptive mechanisms fail, cell death pathways will eventually be induced, eliminating cells beyond repair^89,90,92^.

Rapidly proliferating cancer cells are often subject to ER stress. This is because (1) fast-growing cells place higher demand on ER activity for protein production^93^; (2) inadequate vascularization of tumors leads to hypoxia and nutrition deprivation, which results in inadequate protein glycosylation and ATP production required for proper protein folding^94,95^; and (3) the hypoxic tumor microenvironment leads to disturbances in cellular redox regulation in which oxidizing or reducing agents lead to improper protein folding and the activation of UPR pathways. Indeed, the expression of ER stress markers has been shown to increase in many cancers and has been associated with aggressive phenotypes and therapeutic resistance^96–99^. Furthermore, ER stress was found to increase after treatment with chemotherapeutic drugs in various cancers^100,101^ (**Table 3**).

**Table 3:** List of clinical chemotherapeutic drugs inducing ER stress in cancer cells

Drugs (generic name)	Trade name	Drugs type	Cancer	Reference
Cisplatin	Platinol	Alkylating agent	CRC	^100^
			HCC	^250^
Doxorubicin	Ariamycin PFS/Adriamycin RDF/Rubex	Anthracycline antibiotic	Breast cancer	^251^
Epirubicin	Ellence	Anthracycline antibiotic	Breast cancer	^251^
Gemcitabine	Gemzar	Antimetabolite	PDAC	^252^
Ixabepilone	Ixempra	Plant alkaloid/eopthilones/antimicrotubule agent	RCC	^253^
Paclitaxel	Taxol	Plant alkaloid/taxane/antimicrotubule agent	Breast cancer	^254–256^
			Melanoma	^257^
Vinblastine	Velban	Plant alkaloid	Breast cancer	^254^

CRC, colorectal cancer; HCC, hepatocellular carcinoma; PDAC, pancreatic ductal carcinoma; RCC, renal cell carcinoma.

## UPR and chaperones in chemoresistance

Several mechanisms have been proposed to account for UPR-mediated therapeutic resistance. For example, some pro-survival proteins were found to be induced by UPR signaling. Wroblewski et al.^102^ demonstrated that treatment with the BH3 mimetics Obatoclax and ABT-737 induced anti-apoptotic Bcl-2 family proteins Mcl-2 *via* UPR in melanoma. The drug resistance-related protein prion was also reported to be regulated through UPR pathways in breast cancer^103^.

A major resistance mechanism generated by UPR is through the upregulation of catalysts that accelerate protein folding and molecular chaperones that inhibit protein aggregation under ER stress. These proteins facilitate protein folding, thereby attenuating ER stress and preventing the induction of apoptosis^104^. Downregulation of the ER stress chaperones by small interfering RNA (siRNA) has been shown to increase apoptosis induced by chemotherapeutic drugs^105^. In addition to their function in restoring ER homeostasis, chaperone proteins have also been implicated in several mechanisms of chemoresistance^104,106,107^. For example, protein disulfide isomerase (PDI) is an enzyme that catalyzes disulfide bond formation and isomerization, and is a chaperone facilitating proper folding of many secretory proteins^108^. Inhibition of PDI has been shown to increase apoptosis in response to ER stress^109^. In addition, the overexpression of *PDIA4* and *PDIA6* was found in cisplatin-resistant NSCLC cell lines as well as biopsies from lung cancer patients^110^. The inhibition of PDI4 resulted in the reactivation of the classical mitochondrial pathway, while downregulation of PDIA6 led to a non-canonical cell death pathway with some necroptotic features^110^.

Another major family of chaperones involved in chemoresistance is heat shock proteins (HSPs). HSPs are induced by cell stress and are known to facilitate tumor progression *via* controlling the stability and function of their target proteins^104,111,112^. HSP90 has an indispensable role in regulating mitogenesis and cell cycle progression helping to stabilize fragile structures of receptors, protein kinases, and transcription factors necessary for cell growth^104,113^. For example, HSP90 has been shown to protect the androgen receptor in castration-resistant prostate cancer (CRPC) cells from degradation, and to stabilize multiple components involved in the development and/or maintenance of CRPC including Akt, receptor tyrosine-protein kinase erbB-2 (ERBB2), and cyclin-dependent kinases (CDKs). As a result, blocking HSP90 suppressed the growth and survival signaling of resistant cells, leading to apoptosis and cell cycle arrest^114^. Moreover, the combination of HSP90 inhibitors with other chemotherapeutic drugs has been shown to generate synergistic effects in overcoming chemoresistance both *in vitro* and* in vivo*^115,116^. In a study on mantle cell lymphoma, the combination of the HSP90 inhibitor IPI-504 and bortezomib, an inhibitor of the 26S proteasome, overcame bortezomib resistance by inhibiting UPR and promoting the Noxa-mediated apoptotic pathway^115^. Importantly, in an NSCLC patient with crizotinib resistance, the HSP90 inhibitor ganetespib was able to induce marked tumor shrinkage after one cycle of monotherapy^116^.

HSP27, HSP47, and HSP70 are also involved in cancer drug resistance primarily through preventing apoptosis under cellular stress conditions^111,117,118^. These HSPs have been shown to inhibit proteolytic maturation of caspases, cleavage of their substrate, and apoptosome formation^104,119–121^. Recent studies have demonstrated that OGX-427, a second-generation antisense oligonucleotide, was able to inhibit HSP27 expression both *in vitro* and in xenograft mice *in vivo*^122^. OGX-427 has been shown to suppress tumor progression and enhance the efficacy of gemcitabine, a nucleoside analog chemotherapy, in pancreatic cancer *in vitro* and *in vivo*^122^. Interestingly, many HSPs play overlapping roles in sustaining tumor survival and inhibiting cell death pathways. Simultaneous inhibition of HSP90 and HSP27 has been shown to synergistically increase tumor suppression effects through enhanced apoptosis and ER stress^123,124^. The results of phase II clinical trials using OGX-427 alone for the treatment of CRPC, metastatic NSCLC, pancreatic cancer, and bladder cancer do not look promising, further suggesting that a combinatorial targeting of HSPs may be necessary for effective cancer treatment^125–128^.

GRP78 is a member of the HSP70 superfamily that plays a critical role in cell proliferation, survival, and angiogenesis of various cancers^93^. The induction of GRP78 under ER stress has been regarded as a substantial contributor to chemoresistance in cancer cells^129^. GRP78 has been shown to promote cell survival under therapeutic stress through various mechanisms including through the cytoprotective branches of UPR induction. The expression level of the gene encoding GRP78 is greatly induced under ER stress and like other molecular chaperones, GRP78 prevents the aggregation of misfolded proteins, which can cause toxicity and trigger apoptosis^130^. In addition, GRP78 upregulation has been shown to lead to the stress-dependent activation of p38 and PERK signaling, which promote survival and drug resistance in dormant carcinoma cells^131^. The activation of UPR signaling can also lead to ER stress-associated cell death and as a result, GRP78 induction of UPR is tightly controlled. For example, SPARC (secreted protein acidic and rich in cysteine) was identified as a GRP78-binding partner and was shown to interfere with the association between GRP78 and PERK and the activation of the downstream ER stress signaling in response to chemotherapy^132^. This suggests that a complicated protein network is involved and that further mechanistic studies are required to understand the ER stress-associated chemoresistance.

A subpopulation of GRP78 was found to bind to and inactivate pro-apoptotic proteins to prevent the induction of apoptosis. For example, GRP78 was shown to form a complex with caspase-7 or -12 at the ER surface, preventing their activation and release^133,134^. As capase-12 mediates ER stress-induced apoptosis, inactivation by GRP78 led to resistance of cancer cells to proteasome inhibitors and DNA-damaging agents^135–137^. Moreover, under stress conditions, elevated levels of GRP78 have been shown to suppress cell death signaling by sequestering B-cell lymphoma 2 (BCL-2) from binding with BCL-2-interacting killer (BIK), which is a pro-apoptotic BH3-only protein^138^.

Importantly, the induction of GRP78 by ER stress not only led to an increase in GRP78 in the ER compartment, but also promoted GRP78 relocalization to other cellular locations, including the cell surface, cytosol, mitochondria, nucleus, and the exterior of the cell through secretion^139^. Cell surface GRP78 (sGRP78) was found to act as a co-receptor mediating tumor cell signal transduction, mainly through Akt signaling, to promote cell survival and drug resistance^140–143^. For example, sGRP78 was demonstrated to initiate mitogen-activated protein kinase (MAPK) and AKT-dependent signaling and to downregulate the apoptotic pathway through the binding of active α2-macroglobulin (α2-M*), a serum proteinase inhibitor^144,145^. In prostate cancer, a serum protein complex composed of native α2-M and prostate-specific antigen was shown to bind GRP78, resulting in the activation of mitogen-activated protein kinase kinase 1/2 (MEK1/2), ERK1/2, S6K, and Akt pro-survival pathways and the increase of DNA and protein synthesis^146,147^. Additionally, sGRP78 was also shown to modulate T-cadherin signaling *via* Akt, promoting pro-survival effects in endothelial cells^148^. In addition, sGRP78 can form a complex with oncoprotein cripto to activate MAPK/PI3K signaling and promote tumor cell proliferation^149^. sGRP78 was demonstrated to couple with PI3K, facilitating PIP3 formation and the activation of PI3K/AKT signaling in breast and prostate cancer cells resistant to hormonal therapy^150^.

GRP78 translocation to other cellular location under stress may also promote drug resistance. For instance, mitochondrial GRP78 has been shown to stabilize Raf-1 on the outer membrane of mitochondria, thereby maintaining mitochondrial permeability and preventing the activation of apoptosis under stress^151^. Moreover, a variety of bortezomib-resistant solid tumor cell lines, but not the sensitive myeloma cell lines, were shown to secrete high amounts of GRP78. Secreted GRP78 induced pro-survival signaling by phosphorylation of ERK and suppressed p53-mediated expression of pro-apoptotic Bok and Noxa proteins, leading to bortezomib resistance in endothelial cells^152^. Notably, GRP78 was also induced in non-stressed cells by the treatment with a histone deacetylase (HDAC) inhibitor, which removes the transcription repression exerted by HDAC1 on the ER stress response elements of GRP78, leading to chemoresistance^153,154^.

## UPR and chemoresistance in cancer stem cells (CSCs)

The significance of the stress response and molecular chaperones in stem cell oncogenesis is gradually emerging^155,156^. Several studies indicate that ER stress signaling is involved in the maintenance of stemness properties of CSCs, which is thought to be a mechanism of chemoresistance and tumor recurrence^157–161^. CSCs are a subpopulation of neoplastic cells within a tumor that have an increased ability to seed new tumors upon experimental implantation in appropriate animal hosts^162^. While most chemotherapeutic agents kill the bulk of tumors, CSCs have been shown to survive and proliferate after chemotherapy. For example, DNMT3A-mutant hematopoietic stem cells (HSCs) were reported to exist and expand in the remission samples of patients with acute myeloid leukemia^163^. Similarly, the pool of glioma stem cells has also been shown to expand over time under the exposure to therapeutic doses of temozolomide in both patient-derived and established glioma cell lines^164^.

Although CSCs have been connected with drug inefficiency for years, the exact molecular mechanisms of resistance caused by CSCs are not completely understood. The slow cycling characteristic of CSCs and the overexpression of drug transporters, anti-apoptotic proteins, and DNA damage enzymes only partially explain the entire resistance spectrum^162,165^. There is accumulating evidence showing that the stress response is crucial for sustaining stem-like properties in both normal and neoplastic stem cells, and that the inhibition of ER stress sensors sensitizes sphere-forming cells to apoptosis^166^. van Galen et al.^167^ proposed a model for how stress signaling is integrated within tissue hierarchies and how it coordinates with stemness in HSCs, revealing an indispensable role of UPR in sustaining HSC’s clonal integrity. In addition, the UPR modulator GRP78 has been shown to be necessary for the survival of embryonic stem cell precursors and was also highly expressed in HSCs^168^. Furthermore, the inducible knockout of GRP78 in the hematopoietic system resulted in a significant reduction of HSCs, common lymphoid and myeloid progenitors, and lymphoid cell populations in the mutant mice^169^. Consistent with these findings, UPR has been shown to play an important role in normal stem cells. For example, GRP78 anchored at the plasma membrane has been proposed as a surface marker for CSCs from colon cancer and head and neck squamous cell carcinoma (HNSCC)^170,171^. Knockdown of GRP78 markedly decreased the self-renewal ability and expression of stemness genes, but inversely promoted cell differentiation and apoptosis in CSCs from HNSCC^170^. Moreover, it has been reported that in breast CSCs, GRP78 mediates chemoresistance through β-catenin/ATP-binding cassette super-family G member 2 (ABCG2) signaling^172^. However, as most of the study focused on the association between GRP78 and CSCs, it would be of great importance to know how the individual UPR pathways participate in the regulation of CSC stemness and how it is coordinated with existing stem cell pathways to enable therapeutic targeting of CSCs and chemoresistance.

## UPR and the epithelial-to-mesenchymal transition (EMT) in chemoresistance

Activation of the EMT program in carcinoma cells has been shown to give rise to cells with stem-like properties^173,174^. In human mammary epithelial cells, the induction of EMT resulted in the acquisition of mesenchymal traits and the expression of stem cell markers. Cells undergoing EMT were also shown to form mammospheres, soft agar colonies, and tumors more efficiently^173^. Indeed, EMT activation has been linked to increased anti-apoptotic ability and chemoresistance in various cancers^175–179^. Chemotherapeutic drugs such as cisplatin and doxorubicin have been shown to activate ER stress and subsequent EMT signaling pathways^180^. Interestingly, emerging evidence indicates that there is cross-talk between UPR and EMT activation. UPR induction was shown to potentiate EMT and vice versa^181–183^. Feng et al.^184^ demonstrated that cancer cells undergoing EMT had particularly high sensitivity to ER stress-induced death, suggesting that UPR is constitutively activated in EMT cells. Moreover, EMT gene expression strongly correlated with the PERK-eIF2α axis of UPR and the blocking of PERK signaling pathway interfered with the ability of EMT cells to invade and metastasize. These data suggest that UPR and EMT signaling are closely related and that interfering with the interplay between these two pathways is a potential strategy to suppress CSCs and tumor progression.

## Senescence

Cellular senescence is a status where cells stably exit the cell cycle at the end of the cellular lifespan or in response to different stresses. Senescence is a heterogeneous phenotype. Depending on the type of stimulus, organism of origin, and cellular context, senescent cells may display various senescence markers which contribute to the phenotype to different extents^185^. For example, in addition to a lack of proliferation, senescent cells may show large and flat morphologies, higher senescence-associated β-galactosidase activity (SA-β-Gal), and the appearance of senescence-associated heterochromatin foci (SAHF), a facultative heterochromatin domain that contributes to the silencing of proliferation-promoting genes^186,187^. Although senescent cells remain arrested even when treated with growth factors, these cells are in fact metabolically active and were shown to exert non-cell-autonomous activities by secreting soluble signaling factors. The profound changes in the secretome of senescent cells are termed the senescence-associated secretory phenotype (SASP)^185,188^.

Although it has long been accepted that senescence is a tumor-suppressive mechanism that permanently arrests cells at risk for malignant transformation, accumulating evidence suggests that senescent cells can actually drive tumor progression. They do this through modulation of the tumor microenvironment by altering the secretion of interleukins, inflammatory cytokines, protease, and growth factors in SASP^188^. It has been shown that SASP enhances cell proliferation and motility, regulates tumor immunology, and promotes the emergence of the cancer-stem-like cells^189^. It has also been shown that after cyclic stimulus of senescence-inducing androgen deprivation, senescence-resistant, androgen-refractory cells were generated. These cells were characterized with notable chemoresistance and enhanced pro-survival mechanisms^190^. Several studies also demonstrated that the SASP is associated with therapeutic resistance in the current cancer treatment (**Table 4**). For example, SASP induced by cisplatin administration in melanoma cells may promote the proliferation of non-senescent cells through the activation of ERK1/2–ribosomal S6 kinase 1 (RSK1) pathway^191^. Hepatocellular carcinoma cells resistant to sorafenib were found to be associated with the SASP-related p16/IL6 axis^192^. Interestingly, Mastri et al.^193^ found a temporary cellular change similar to SASP after the withdrawal of anti-angiogenic therapy (SASP-mimicking anti-angiogenic therapy-induced secretome, ATIS) in cancer cells. The senescence hallmarks observed in these cells ultimately reversed after long drug withdrawal periods. This incomplete induction of the SASP phenomenon may explain the highly diverse treatment efficacy observed in patients receiving anti-angiogenic therapy^193^.

**Table 4:** List of clinical chemotherapeutic drugs inducing SASP in cancer cells

Drug (generic name)	Trade name	Drugs type	Cancer	Reference
Cisplatin	Platinol	Alkylating agent	Melanoma	^191^
Doxorubicin	Ariamycin PFS/Adriamycin RDF/Rubex	Anthracycline antibiotic	CRC	^258^
Gemcitabine	Gemzar	Antimetabolite	PDAC	^259^
Temozolomide	Temodar/Temodal/Temcad	Alkylating agent	Melanoma	^260^

CRC, colorectal cancer; PDAC, pancreatic ductal carcinoma.

Importantly, resistance induced by the SASP was also observed in clinics. Malignant pleural mesothelioma (MPM) patients with upregulated senescence marker were shown to have a worse prognosis after receiving platinum-based therapy^194^. To explore the potential mechanism by which senescence induces chemoresistance, Canino et al.^195^ used MPM as a model and found that conditioned media from pemetrexed-treated senescent MPM cells induced the emergence of EMT-like, clonogenic, and chemoresistant cell subpopulations with high levels of aldehyde dehydrogenase (ALDH) activity. It was shown that these SASP-cytokine-induced chemoresistant cells could be targeted by STAT3 knockdown or HSP90 inhibition, resulting in a reduction of the population of high ADLH-expressing cells and EMT genes expression both *in vitro* and *in vivo*^195,196^. This suggests that SASP signaling-mediated pathways may be a potential target in anti-cancer therapy.

## Conclusion

**Figure 2:**
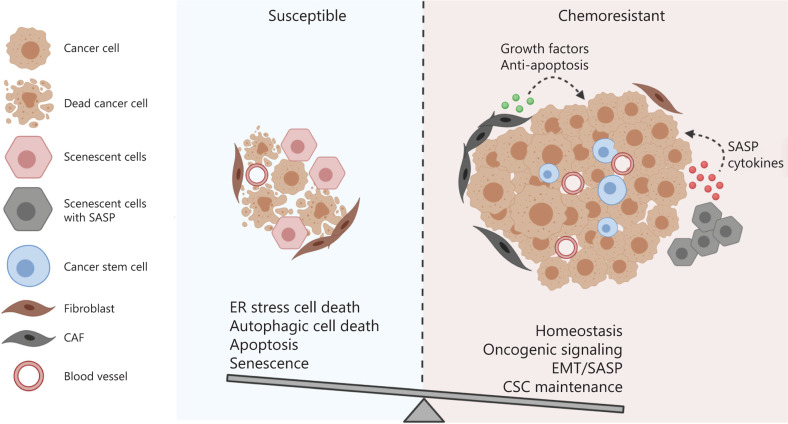
The balance between the integrated cell survival and death signaling determines the cell fate of cancer cells receiving chemotherapy. Multiple cellular adaptive response may be induced in cancer cells following chemotherapy. The cell fate is determined by the balance between the integrated pro-survival and pro-death adaptive response. If the pro-survival signaling overrides the death-triggering signaling, the cancer cells are resistant to the treatment and may continue to proliferate and advance in the patients.

The adaptive response is essential for cell and organism survival of sublethal cellular damage and disruption of homeostasis. The transient and reversible adjustments in response to stress can be mediated by biochemical or post-translational mechanisms, or rely on alterations in gene expression^197^. The activation of autophagy and ER stress signaling have been shown to play important roles in the restoration of cellular homeostasis, while senescence may contribute to growth arrest, thereby extending the life span of an organism under stress. Nevertheless, as discussed already, these adaptive responses may in turn promote resistance to chemotherapy (**Figure 2**). One of the common characteristics of autophagy and ER stress signaling is that their activation can either lead to cell survival or death, depending on the levels and types of stress. However, the specific factors that activate the death program still remain to be elucidated in both autophagy and ER stress signaling. Several mechanisms have been proposed to be involved, and cell signaling pathways may interact in different ways to have specific outcomes in different cell types^91,198^. This may partly explain the variable efficacy of bortezomib and hydroxychloroquine treatment in cancer patients^199,200^. Therefore, understanding how cancer cells integrate information from different signaling pathways, and the establishment of a system quantitatively monitoring the stress intensity and duration and its subsequent effects on cell fate following chemotherapeutic drugs administration, are particularly needed to overcome chemoresistance. Tumor heterogeneity and the influence of the tumor microenvironment may pose a challenge for establishing a standard treatment protocol. However, due to the recent advances in computational biology and single-cell multi-omics approaches, this may be attainable^201–203^. Collaboration between oncologists, cancer biologists, and bioinformaticians is necessary to overcome the immense challenge of chemotherapy resistance.
